# SIGIRR-caspase-8 signaling mediates endothelial apoptosis in Kawasaki disease

**DOI:** 10.1186/s13052-022-01401-8

**Published:** 2023-01-04

**Authors:** Zhengwang Wen, Yuhan Xia, Yingying Zhang, Yuxi He, Chao Niu, Rongzhou Wu, Chunxiang Zhang, Chang Jia, Xing Rong, Maoping Chu

**Affiliations:** 1 Children’s Heart Center, Institute of Cardiovascular Development and Translational Medicine, The Second Affiliated Hospital and Yuying Children’s Hospital of Wenzhou Medical University, 325027 Wenzhou, China; 2 Pediatric Research Institute, The Second Affiliated Hospital and Yuying Children’s Hospital of Wenzhou Medical University, Wenzhou, 325000 China; 3 Key Laboratory of Structural Malformations in Children of Zhejiang Province, Wenzhou, 325000 Zhejiang Province China

**Keywords:** Kawasaki disease, Endothelial cell, SIGIRR, Caspase-8, Apoptosis

## Abstract

**Background:**

Kawasaki disease (KD) is a kind of vasculitis with unidentified etiology. Given that the current diagnosis and therapeutic strategy of KD are mainly dependent on clinical experiences, further research to explore its pathological mechanisms is warranted.

**Methods:**

Enzyme linked immunosorbent assay (ELISA) was used to measure the serum levels of SIGIRR, TLR4 and caspase-8. Western blotting was applied to determine protein levels, and flow cytometry was utilized to analyze cell apoptosis. Hematoxylin eosin (HE) staining and TUNEL staining were respectively used to observe coronary artery inflammation and DNA fragmentation.

**Results:**

In this study, we found the level of SIGIRR was downregulated in KD serum and KD serum-treated endothelial cells. However, the level of caspase-8 was increased in serum from KD patients compared with healthy control (HC). Therefore, we hypothesized that SIGIRR-caspase-8 signaling may play an essential role in KD pathophysiology. In vitro experiments demonstrated that endothelial cell apoptosis in the setting of KD was associated with caspase-8 activation, and SIGIRR overexpression alleviated endothelial cell apoptosis via inhibiting caspase-8 activation. These findings were also recapitulated in the *Candida albicans* cell wall extracts (CAWS)-induced KD mouse model.

**Conclusion:**

Our data suggest that endothelial cell apoptosis mediated by SIGIRR-caspase-8 signaling plays a crucial role in coronary endothelial damage, providing potential targets to treat KD.

## Introduction

Kawasaki disease (KD) is an acute systemic vasculitis that mainly occurs in children below the age of five. The etiology and pathogenesis of KD are still unknown and the main complication is coronary artery injury [1, 2]. Administration of intravenous immunoglobulin (IVIG) and aspirin has greatly decreased the incidence of coronary lesions in KD patients. However, the therapeutic effect of this strategy is not ideal in the face of complicated or refractory cases. Therefore, there is an urgent need to unravel the potential pathogenesis mechanisms of KD and explore novel and effective therapeutic options.

Endothelial cell injury is one of essential pathological mechanisms in KD, targeting which is a good candidate to prevent and cure KD coronary artery injury [3–6]. As is known, toll-like receptors (TLRs) can mediate the pro-inflammation response, and excessive and dysfunctional TLR signaling may lead to severe inflammation and inappropriate tissue damage [7, 8]. Studies reported that the expression levels of toll-like receptor 4 (TLR4) are increased during acute phase of KD compared with healthy controls, indicating that TLR4 might be involved in the vascular inflammation in KD patients [9, 10]. In addition, studies substantiated that single immunoglobulin IL-1R-related receptor/IL-1 receptor 8 (SIGIRR/IL-1R8) is a member of the toll-interleukin-1 receptor (TIR) family that can negatively regulate TLR4-mediated signaling. For instance, Liu et al. reported that ectopic expression of SIGIRR in the colon ameliorates colitis in mice by inhibiting TLR4/NF-κB overactivation [11]. Our previous studies have demonstrated that IL-37b mitigates endothelial cell apoptosis and inflammation in KD through SIGIRR [5], which indicates that SIGIRR plays a protective role in endothelial cell injury in KD. However, the exact role and mechanism of SIGIRR involved in KD vasculitis merit a further investigation.

In this study, we first determined the levels of SIGIRR, TLR4 and caspase-8 in the serum from healthy controls (HCs), ordinary febrile patients, acute KD patients, and convalescent phase of KD patients. In addition, we also analyzed the protein expression of SIGIRR and TLR4 in endothelial cells after treatment with the above-mentioned sera. Then, in vitro experiments investigated the protective role and mechanisms of SIGIRR, which were also substantiated in a murine model of KD induced by *Candida albicans* cell wall extracts (CAWS).

## Materials and methods

### Collection of blood samples and ethical consideration

Blood samples were collected from KD patients, age-matched healthy controls (HCs), ordinary febrile children (Febrile) and convalescent phase—KD patients (Conv KD). These subjects went to the Second Affiliated Hospital and Yuying Children’s Hospital of Wenzhou Medical University between November 2019 to October 2021. The age range of HCs, Febrile, KD and Conv KD was respectively 48.4 ± 21.03, 46.0 ± 13.67, 47.5 ± 17.74 and 44.36 ± 11.35 months. There was no statistical difference about age and gender among these groups. Healthy controls (HCs) were healthy physical examinees. Patients diagnosed with KD met the criteria set by the American Heart Association in 2017. Febrile children were patients with fever for at least three days accompanying by no KD criteria. Convalescent samples were subjects whose acute illness was resolved and maintained for at least one month. All KD patients received intravenous immunoglobulin (IVIG, 2 g/kg) plus aspirin (30-50 mg/kg/d) during the acute phase. The sera were stored at -80℃ within 4 h after collection until later use. All the participants signed consent forms for participation in the academic research. This study was authorized by the Ethics Committee of The Second Affiliated Hospital and Yuying Children’s Hospital of Wenzhou Medical University (LCKY2019-47) and was conducted in accordance with the Declaration of Helsinki.

### Enzyme-Linked Immunosorbent Assay (ELISA)

Serum levels of SIGIRR, TLR4 and caspase-8 were respectively examined by the corresponding ELISA kits according to the manufacturer’s instructions. These ELISA kits were purchased from ProteinTech Group (Chicago, USA).

### Cell culture and treatment

Human umbilical vein endothelial cells (HUVECs) were routinely cultured in high glucose Dulbecco’s modified Eagle’s medium (DMEM) supplemented with 10% fetal bovine serum (FBS), and 1% (v/v) pencillin/streptomycin at 37 °C with 5% CO_2_/95% air. HUVECs were placed in 6-well plates (Becton Dickinson Labware, Franklin Lakes, NJ, USA) for protein extraction or apoptosis analysis. These endothelial cells were respectively treated by 10% HC serum, Febrile serum, KD serum and Conv KD serum. If necessary, the HUVECs would be pretreated with inhibitors for 60 min, and then treated with KD serum or HC serum.

### Western blot analysis

Total proteins were extracted from treated endothelial cells or heart tissues, and then protein concentrations were determined by BCA assay. Next, equal amounts of proteins (60 μg) were separated by 10% SDS-PAGE gel and electro-transferred to nitrocellulose membranes. After a 2-h block with 5% skimmed milk, nitrocellulose membranes were incubated with the following primary antibodies at 4 °C overnight: SIGIRR (Proteintech, Chicago, USA, 1:500, Cat. No.:27828–1-AP), TLR4 (Proteintech, Chicago, USA, 1:1000, Cat. No.:19811–1-AP), cleaved caspase-8 (Proteintech, Chicago, USA, 1:500, Cat. No.:66093–1-lg), cleaved caspase-3 (ABclonal, Boston, USA, 1:500, Cat. No.:A19654), Bcl-2 (Abcam, Cambridge, UK, 1:500, Cat. No.: ab182858), BAX (Abcam, Cambridge, UK, 1:1000, Cat. No.: ab182733), GAPDH (Proteintech, Chicago, USA, 1:2000, Cat. No: 60004–1-lg). After that, the membranes were washed with TBST buffer, and then incubated with secondary antibodies conjugated with horseradish peroxidase (HRP) (1:1000) for 2 h. Signals were visualized, and the densitometric values of bands were obtained by the Image J software and then were subjected to statistical analysis.

### Endothelial cell apoptosis determination

After endothelial cells were treated with the indicated serum and/or reagents, cells were collected and washed with PBS for two times. Next, the cells were resuspended in 500 μl flow cytometry staining buffer with the addition of 5 μl Annexin-FITC and 10 μl PI. After that, the stained cells were analyzed by flow cytometry (Becton Dickinson, USA).

### Preparation of *Candida albicans* cell wall extracts (CAWS)

Studies reported that CAWS can induce coronary arteritis in mice that resembles KD. Therefore, CAWS-induced coronary arteritis is regarded as a good model for investigating the pathology of arteritis [12, 13]. In this study, CAWS were obtained according to previously described methods [5, 6, 14]. Briefly, *Candida albicans* strain NBRC1385 were grown in C-limiting medium at 27 °C for 2 days at a rotation speed of 270 rpm. Then an equal volume of ethanol was added and placed in a refrigerator at 4 °C overnight. After that, the cultures were centrifuged to obtain the pellets, which were then mixed with an equal amount of water with stirring for 2 h. After centrifugation again, the supernatant was collected and mixed again with ethanol at 4 °C overnight. Finally, the complex was centrifuged, and the precipitate was acquired by dry with acetone. The obtained CAWS were dissolved in PBS buffer and autoclaved before use.

### Animals and ethics statements

Male C57BL/6 mice (3–4 weeks) were used to create mouse models of KD, which were obtained from Wenzhou Medical University, License No. SYXK[ZJ]2015–0009. All experimental procedures involved in animal studies were authorized by the ethics committee of Wenzhou Medical University (wydw2020-0587) and conducted according to the Guide for the Care and Use of Laboratory Animals. Mice were randomly divided into five groups (n = 6 for each group), including PBS group, CAWS group, CAWS + AAV-empty group, CAWS + AAV-SIGIRR group, and CAWS + AAV-SIGIRR + DHC group. Mice in the PBS group were injected with PBS buffer, while mice used for KD induction were intraperitoneally injected with CAWS (4 mg/body) once a day for 5 days. AAV virus was injected through tail vein at a dose of 2 × 10^12^ vg per mice before one week of the first CAWS administration. After 4 weeks of final CAWS injection, mice were anesthetized and sacrificed for collection of heart tissues and follow-up examinations.

### Hematoxylin and Eosin (H&E) Staining and immunofluorescence staining

Mice heart tissues were collected, fixed with 4% paraformaldehyde and embedded with paraffin. 5-µm-thick heart sections were dewaxed and hydrated, and then stained with H&E solution. After that, the stained sections were observed under light microscope. TUNEL staining was conducted as described before. DAPI was used to indicate the nucleus, and CD31 was utilized to mark the endothelial cells [15, 16]. Finally, the images were captured under confocal laser microscopy (Nikon, Tokyo, Japan).

### Data analysis

Every experiment was performed at least three times, and data were presented as mean ± SD. All the results were analyzed using SPSS version 17.0. For the comparison of two experimental groups, a two-tailed unpaired Student’s t test was performed. For more than two groups, one-way analysis of variance test followed by Duncan’s multiple range test was conducted. *P* values < 0.05 was regarded as statistically signficant.

## Results

### Levels of TLR4, SIGIRR and caspase-8 are increased in KD serum and KD serum-treated endothelial cells

**Fig. 1:**
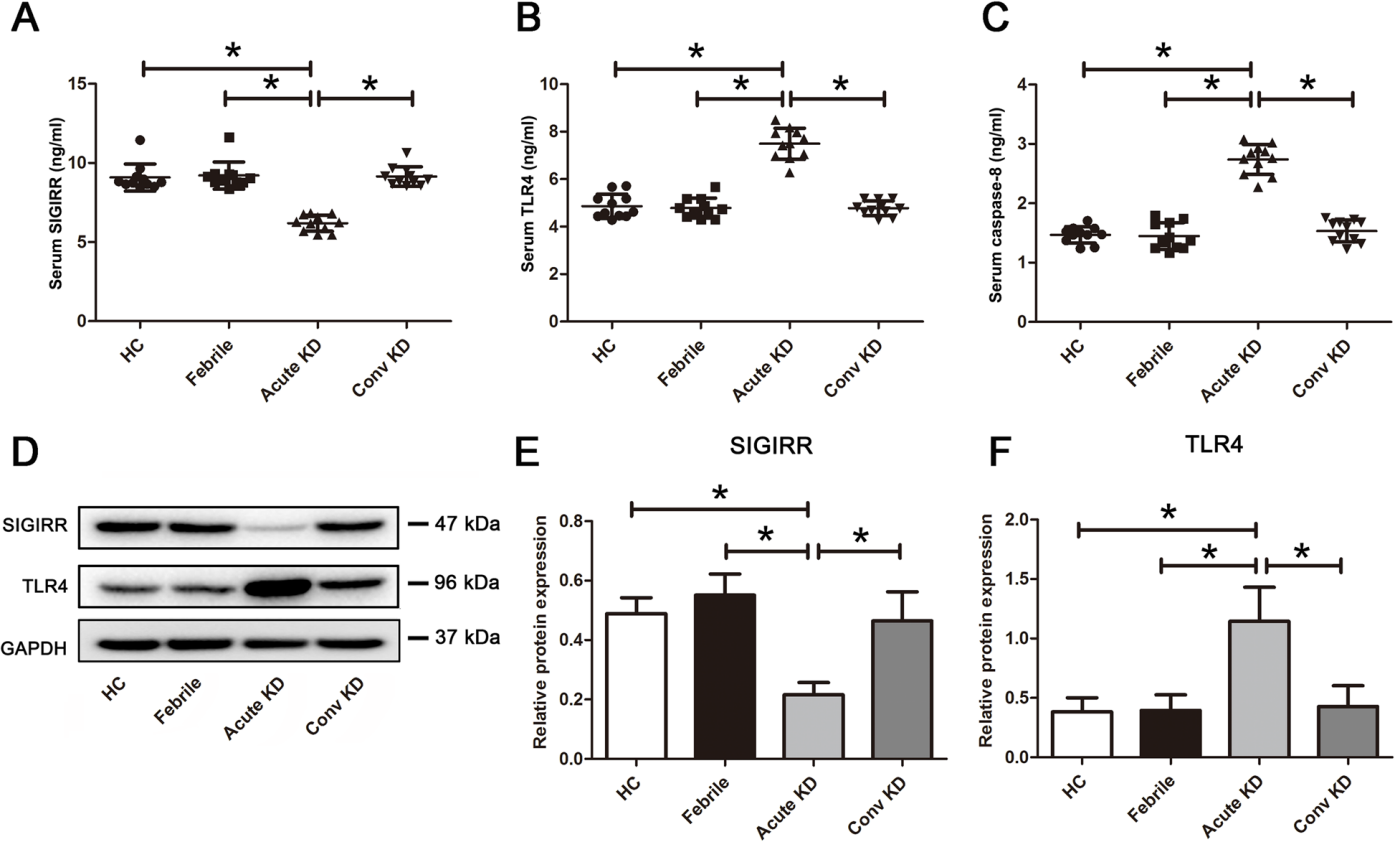
Levels of SIGIRR, TLR4 and/or caspase-8 in KD serum and KD serum-treated endothelial cells were elevated. **A**-**C** The levels of SIGIRR, TLR4 and caspase-8 were measured in the sera from HC, Febrile, Acute KD and Conv KD by ELISA (*n* = 11). **D**-**F** The protein expression of SIGIRR and TLR4 were determined in the treated endothelial cells by Western blot analysis. Data were shown as mean ± SD (*n* = 3). * *P* < 0.05 indicated the significant difference

Previous studies reported that endothelial cell apoptosis is involved in KD pathophysiological process [17]. It is known that TLR4 can mediate cell apoptotic process via initiating caspase-8 activation [17, 18], and caspase-8 is a critical component of death receptor-mediated apoptosis [19]. SIGIRR, as a negative regulator of TLR4 signaling, might be able to suppress apoptosis through inhibiting TLR4. Therefore, to preliminarily assess whether these three proteins were involved in KD, the serum levels of SIGIRR, TLR4 and caspase-8 were examined. Results showed that the levels of SIGIRR were significantly decreased, but TLR4 and caspase-8 were remarkably increased in sera from KD subjects compared with HC, Febrile and Conv KD children (Fig. 1A-C). Western blot analysis displayed that the protein expression of SIGIRR was down-regulated, but TLR4 expression was up-regulated in human umbilical vein endothelial cells (HUVECs) after treatment with KD serum (Fig. 1D-F) compared with other groups. These results indicated that SIGIRR, TLR4 and caspase-8 might participate in KD endothelial cell apoptosis.

### Endothelial cell apoptosis is associated with caspase-8 activation

**Fig. 2:**
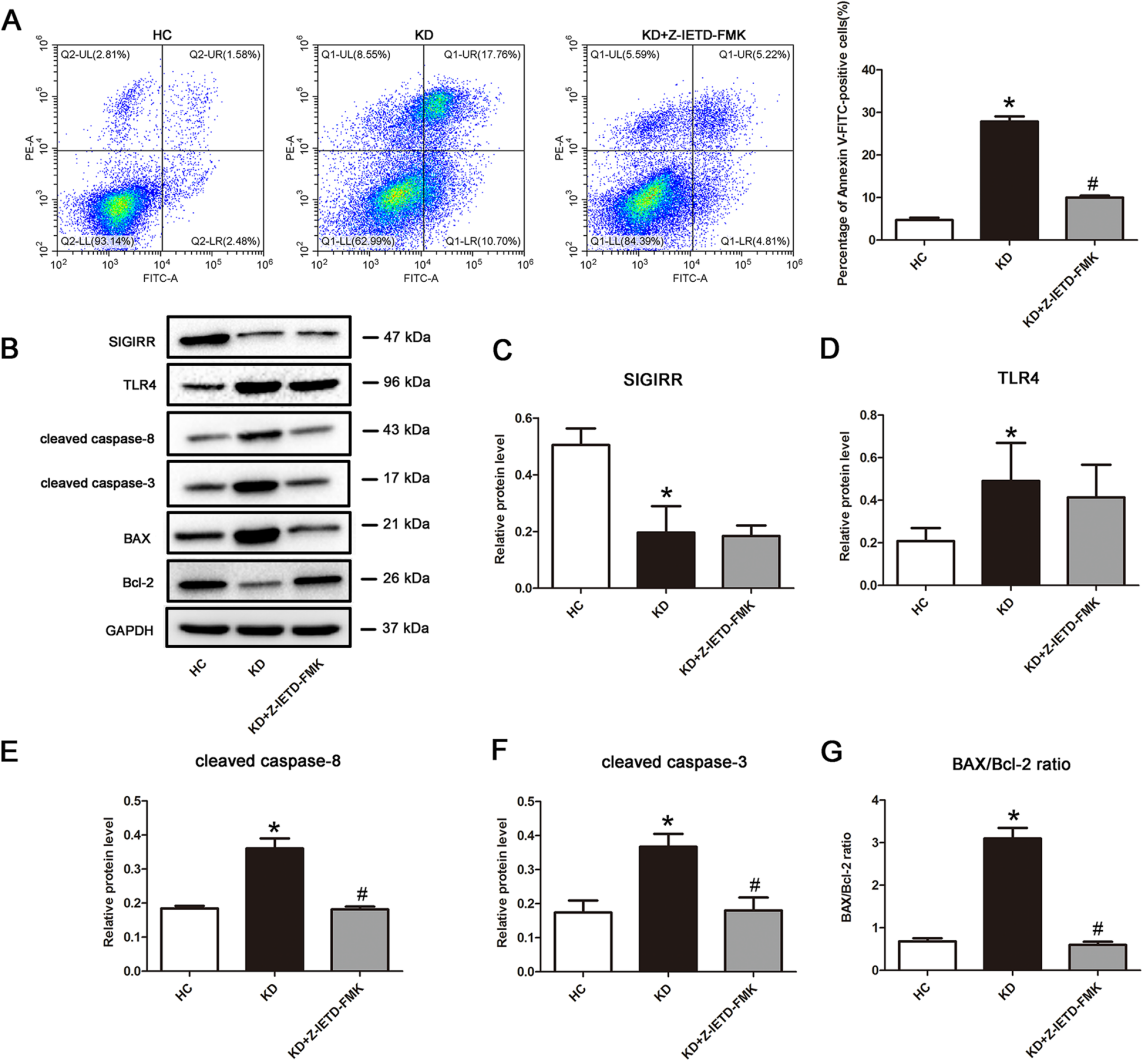
Endothelial cell apoptosis in KD condition was associated with caspase-8 activation. **A** The apoptotic percentage of endothelial cells was analyzed using flow cytometry. The histogram was the quantitative analysis of Annexin V-FITC-positive cells. **B**-**G** The protein expression levels of SIGIRR, TLR4, cleaved caspase-8 and cleaved caspase-3, and BAX/Bcl-2 ratio were determined in the treated endothelial cells by Western blotting. Data were presented as mean ± SD (*n* = 3). * *P* < 0.05 versus HC group, and # *P* < 0.05 versus KD group

As is known, caspase-8 plays an important role in death receptor-mediated apoptosis [20, 21]. To confirm whether endothelial cell apoptosis was regulated by casapase-8, caspase-8 activity was inhibited using a caspase-8-specific inhibitor, Z-IETD-FMK [22]. Results exhibited that addition of Z-IETD-FMK significantly reduced the percentage of apoptotic endothelial cells compared with KD group (Fig. 2A). Further study showed that inhibition of caspase-8 activity remarkably decreased the expression of downstream proteins, including cleaved caspase-8 and cleaved caspase-3. In addition, the ratio of BAX/Bcl-2 was also declined by caspase-8 inhibition. However, the protein expression of SIGIRR and TLR4 were not affected by the addition of Z-IETD-FMK (Fig. 2B-G), suggesting that SIGIRR and TLR4 might locate upstream of caspase-8. Together, these data revealed that caspase-8 mediated KD serum-induced endothelial cell apoptosis.

### Overexpression of SIGIRR alleviates KD serum-induced endothelial cell apoptosis

**Fig. 3:**
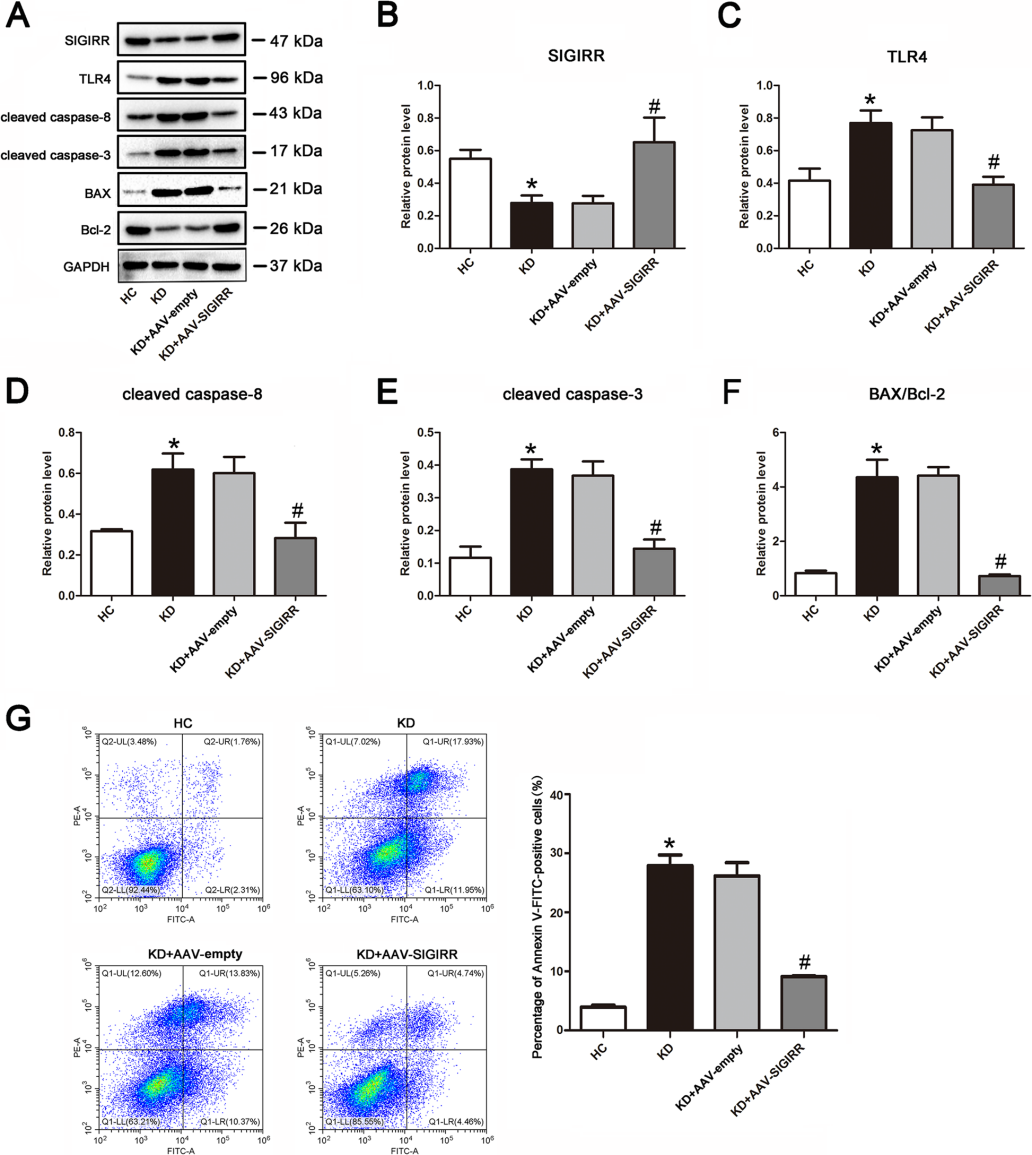
SIGIRR overexpression rescued endothelial cell apoptosis. **A**-**F** The protein expression levels of SIGIRR, TLR4, cleaved caspase-8 and cleaved caspase-3, and BAX/Bcl-2 ratio were determined in the treated endothelial cells by Western blotting. **G** The apoptotic percentage of endothelial cells was analyzed using flow cytometry. The histogram was the quantitative analysis of Annexin V-FITC-positive cells. Data were expressed as mean ± SD (*n* = 3).* *P* < 0.05 versus HC group, and # *P* < 0.05 versus KD group

As is known, SIGIRR can regulate cell apoptosis [5]. Above findings have demonstrated that SIGIRR expression was down-regulated in endothelial cells upon KD serum treatment. To investigate its role, SIGIRR was overexpressed in endothelial cells before treatment with KD serum. Results exhibited that SIGIRR overexpression notably decreased the protein expression levels of TLR4, cleaved caspase-8 and cleaved caspase-3, and the ratio of BAX/Bcl-2 compared with KD group, revealing that SIGIRR could alleviate KD serum-induced apoptosis, and it was localized upstream of these proteins (Fig. 3A-F). Further study displayed that the percentage of apoptotic endothelial cells was also significantly reduced by SIGIRR overexpression compared with KD group (Fig. 3G). Taken together, these observations suggest that SIGIRR could mitigate endothelial cell apoptosis in KD conditions.

### SIGIRR suppresses endothelial cell apoptosis via inhibiting caspase-8 activation

**Fig. 4:**
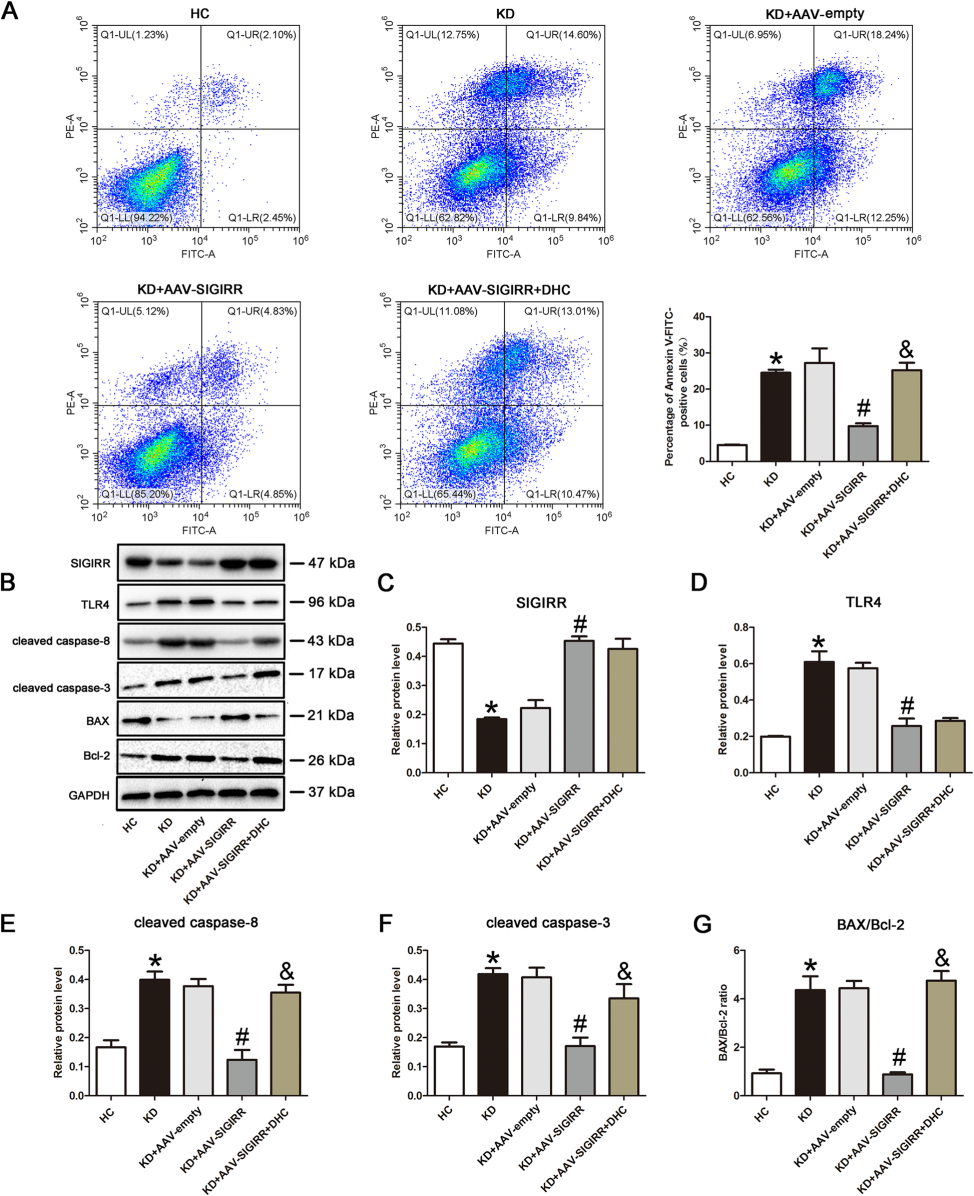
SIGIRR mitigated endothelial cell apoptosis via inhibiting caspase-8 activity. **A** The apoptotic percentage of endothelial cells was analyzed using flow cytometry. **B**-**G** The protein expression levels of SIGIRR, TLR4, cleaved caspase-8 and cleaved caspase-3, and BAX/Bcl-2 ratio were determined in the treated endothelial cells by Western blotting. * *P* < 0.05 versus HC group, # *P* < 0.05 versus KD group, and & *P* < 0.05 versus KD + AAV-SIGIRR group

We substantiated that SIGIRR could regulate cleaved caspase-8 expression, but inhibition of caspase-8 activity had no effect on SIGIRR expression, indicating that SIGIRR should be located upstream of caspase-8. Therefore, we hypothesized that SIGIRR might ameliorate KD serum-induced apoptosis through suppressing caspase-8 activity. To confirm it, we simultaneously overexpressed SIGIRR and activated caspase-8. As expected, activation of caspase-8 remarkably reversed SIGIRR-mediated decrease in the percentage of apoptotic endothelial cells (Fig. 4A). Moreover, the decreased protein levels of cleaved caspase-8, cleaved caspase-3, and BAX/Bcl-2 ratio by SIGIRR overexpression were also increased by caspase-8 activation (Fig. 4B-G). These data manifested that SIGIRR inhibited cell apoptosis through suppressing caspase-8 activation.

### SIGIRR mitigates coronary inflammation and endothelial cell apoptosis in a murine model of KD

**Fig. 5:**
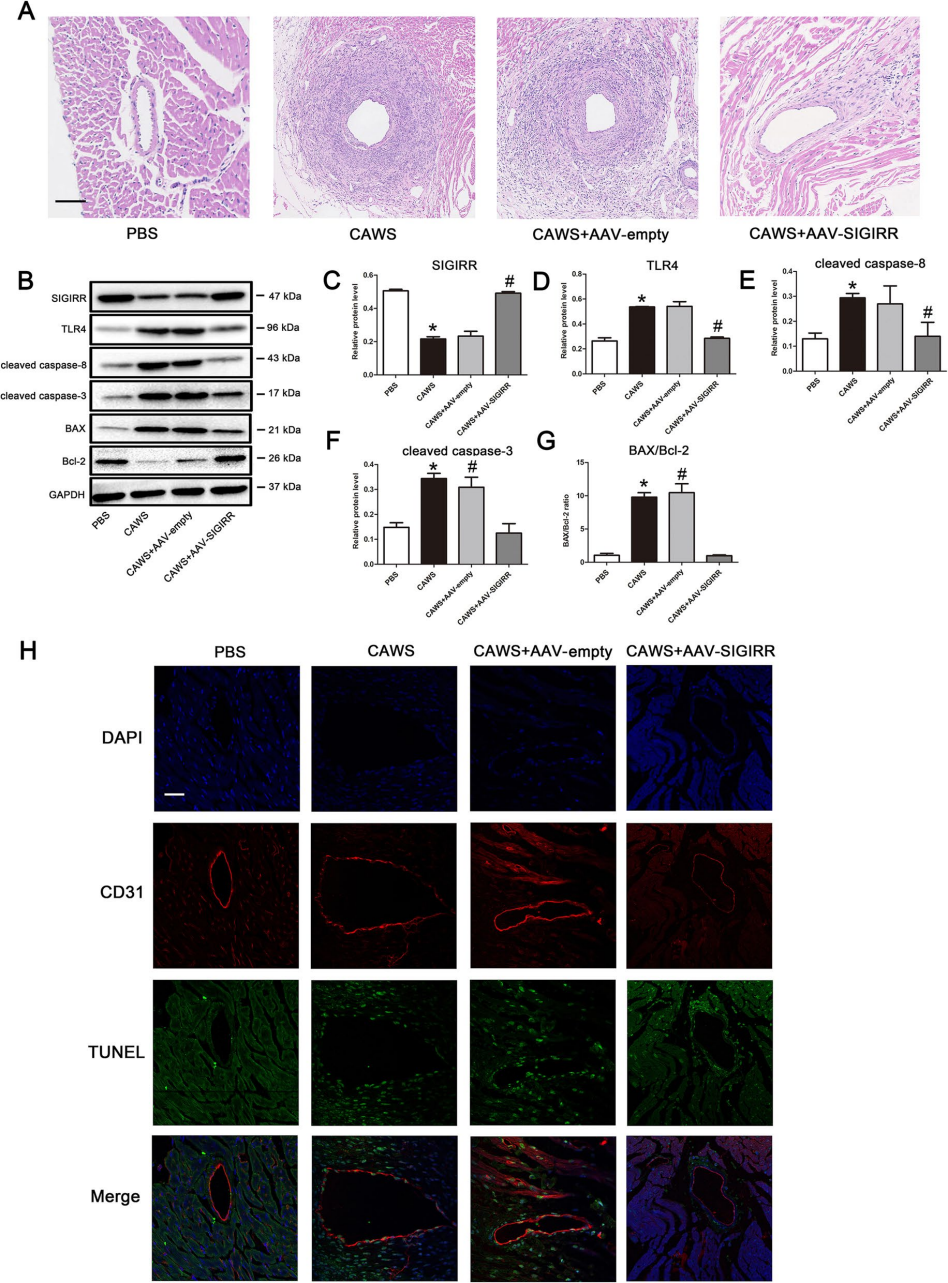
SIGIRR overexpression alleviated coronary artery injury in a murine model of KD. **A** Heart tissues were harvested on 28 day after CAWS injection, and the representative images from H&E staining were shown. Magnification: × 200. Scale bar = 100 μm. **B**-**G** The protein expression of SIGIRR, TLR4, cleaved caspase-8 and cleaved caspase-3, and BAX/Bcl-2 ratio in the heart tissues were determined by Western blotting. **H** DNA fragmentation in the endothelial cells was assessed by co-localized staining of TUNEL (green) and CD31 (an endothelial marker, red) (*n* = 5). Magnification: × 200. Scale bar = 50 μm. * *P* < 0.05 versus PBS group, and # *P* < 0.05 versus CAWS group

To in vivo substantiate the protective role of SIGIRR, an adeno-associated virus 9 (AAV9) -mediated SIGIRR overexpression was administrated through the tail vein into KD mice, and AAV9-empty was used as a control. Results showed that SIGIRR overexpression significantly alleviated coronary artery inflammation (Fig. 5A), decreased the expression levels of TLR4, cleaved caspase-8 and cleaved caspase-3, and BAX/Bcl-2 ratio (Fig. 5B-G). Further study exhibited that the percentage of TUNEL-positive cells in the endothelium of coronary artery was notably declined upon overexpression of SIGIRR (Fig. 5H), indicating that SIGIRR played a protective role against KD coronary arteritis via inhibiting inflammation and endothelial cell apoptosis.

### SIGIRR relieves coronary endothelial injury via inhibiting caspase-8 activation in the KD mouse model

**Fig. 6:**
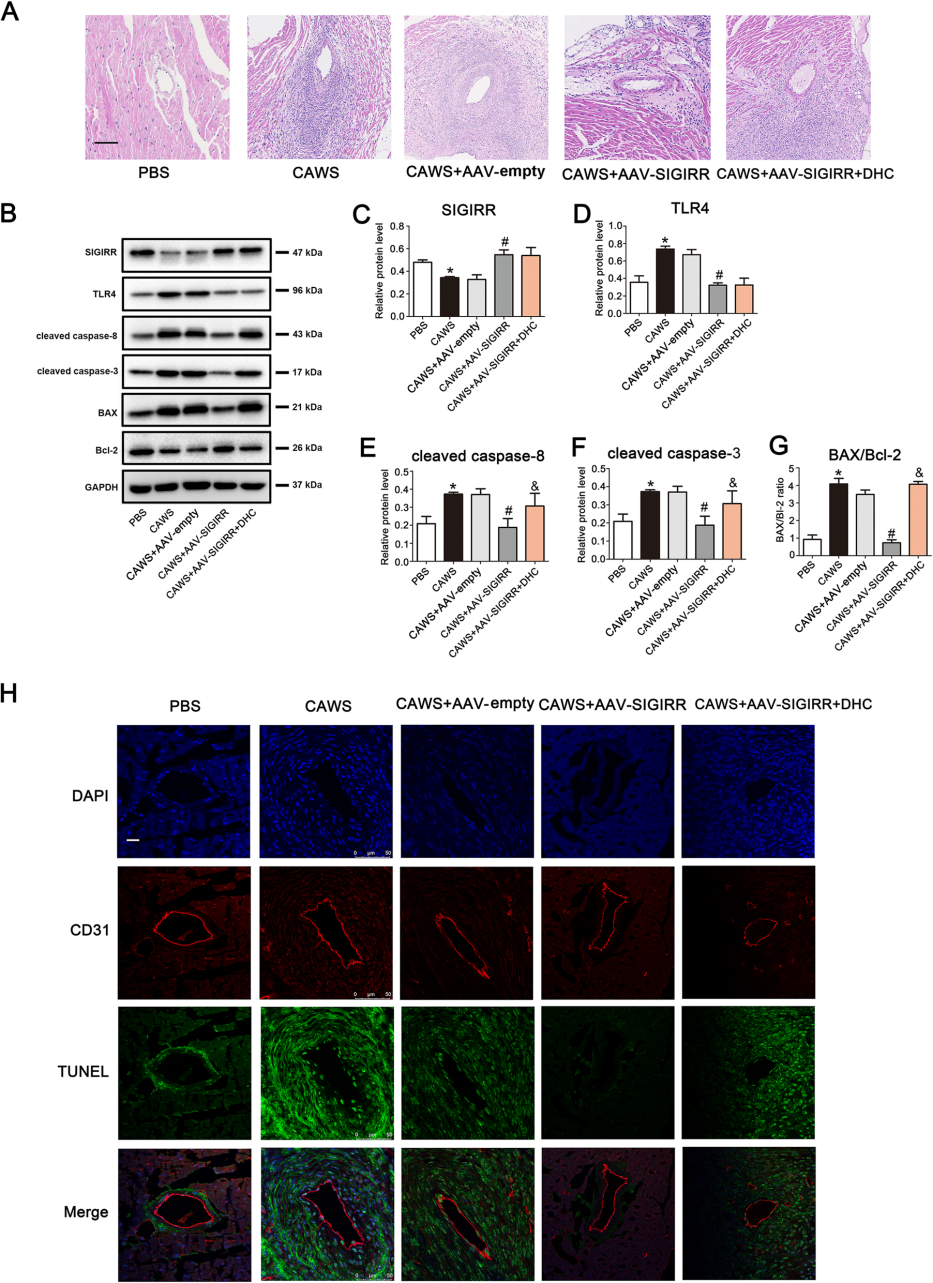
SIGIRR relieved coronary artery injury via inhibiting caspase-8 activation. **A** The representative images from H&E staining were shown. Magnification: × 200. Scale bar = 100 μm. **B**-**G** The protein expression of SIGIRR, TLR4, cleaved caspase-8 and cleaved caspase-3, and BAX/Bcl-2 ratio in the heart tissues were detected by Western blotting. **H** DNA fragmentation in the coronary endothelium was evaluated by co-localized staining of TUNEL (green) and CD31 (an endothelial marker, red) (*n* = 5). Magnification: × 200. Scale bar = 50 μm. * *P* < 0.05 versus PBS group, # *P* < 0.05 versus CAWS group, and & *P* < 0.05 versus CAWS + AAV-SIGIRR group

To in vivo demonstrate whether SIGIRR contributed to anti-inflammation and anti-apoptosis roles via suppressing caspase-8 activity in the KD mouse model, we activated caspase-8 in SIGIRR overexpression mice using caspase-8 agonist – dehydrocorydaline (DHC) [23]. As anticipated, caspase-8 activation aggravated coronary artery inflammation compared with the SIGIRR overexpression group (Fig. 6A). In addition, the expression of cleaved caspase-8 and cleaved caspase-3, and BAX/Bcl-2 ratio was up-regulated after caspase-8 activation (Fig. 6B, E–G). However, the expression of SIGIRR and TLR4 was not affected by activating caspase-8 (Fig. 6B-D). Moreover, SIGIRR-mediated decrease in the percentage of TUNEL-positive cells in the endothelium of coronary artery was also reversed by caspase-8 activation (Fig. 6H). These data suggest that SIGIRR alleviates coronary endothelium damage via inhibiting caspase-8 activity.

## Discussion

Kawasaki disease is the archetypal pediatric vasculitis of unknown etiology, with predilection for coronary arteries. Owing to lack of a reliable confirmatory laboratory test, the current diagnosis of KD is mainly based on a constellation of clinical findings, which have been modified from time to time [24]. Considering the incomplete or atypical forms of KD and IVIG-resistant KD, exploring the pathogenesis of KD, and searching for specific biomarker and therapeutic targets appear to be of particular importance.

TLR4 can regulate both infection-induced or sterile inflammation by endogenous molecules, and apoptotic process [25–27]. In our study, TLR4 levels were increased in serum from KD patients and KD serum-treated endothelial cells. Several studies reported that TLR4 can activate caspase-8, which is very important for death receptor-mediated apoptosis [19, 28, 29]. Moreover, our data also demonstrated that the levels of caspase-8 in KD serum were also increased, revealing caspase-8-mediated apoptosis might occur in KD. To confirm this hypothesis, we inhibited caspase-8 activity, and found that inhibition of caspase-8 significantly rescued KD serum-induced endothelial cell apoptosis, and reduced the expression of apoptosis-related proteins, indicating caspase-8 activation participated in KD serum-mediated apoptosis. Since caspase-8 inhibition had no effect on the expression of SIGIRR and TLR4, indicating that these two proteins might locate upstream of caspase-8.

Studies reported that SIGIRR exhibits broad anti-inflammatory properties in inflammatory diseases, such as acute lung inflammation [30], and can inhibit TLR4 signaling pathway via its Toll-interleukin-1 receptor (TIR) domain [11, 31, 32]. Moreover, as a negative regulator of IL-1 receptor and Toll-like receptor signaling, the levels of SIGIRR were demonstrated to be decreased in KD serum and KD serum-treated endothelial cells, implying that the pathological mechanisms of KD might be associated with SIGIRR. To further investigate the role of SIGIRR, this protein was overexpressed in endothelial cells. As anticipated, overexpression of SIGIRR significantly decreased the expression levels of TLR4, cleaved caspase-8 and other apoptosis-associated proteins. Moreover, the percentage of apoptotic endothelial cells was notably reduced by SIGIRR overexpression compared with KD group, indicating that SIGIRR could suppress KD serum-induced endothelial cell apoptosis. Our further study demonstrated that SIGIRR rescued endothelial cell apoptosis via inhibiting caspase-8 activation. Previous studies reported that TLR4 signaling plays a predominant role in the innate immune response of retinal ischemia by promoting the activation of caspase-8 signaling, and inhibition of caspase-8 has no influence on the production of TLR4 in IOP-induced retinal injury, indicating that TLR4 signaling contributes to the caspase-8 activation [28]. It is known that SIGIRR, as a negative regulator of TLR4, can down-regulate TLR4-mediated signaling [11, 33], further substantiating that SIGIRR could indeed modulate caspase-8 activity, possibly via TLR4.

To in vivo further substantiate the protective effects and mechanisms of SIGIRR, this protein was overexpressed in a KD mouse model. As expected, SIGIRR overexpression significantly alleviated coronary artery inflammation, and reduced the percentage of apoptotic cells in the endothelium of coronary artery. Furthermore, the expression of TLR4 and apoptosis-related proteins was down-regulate upon overexpression of SIGIRR. Li et al. demonstrated that the deubiquitinase USP13 inhibits lung inflammation via stabilizing the anti-inflammatory receptor Sigirr [30]. Riva et al. showed that SIGIRR is an interleukin-1 receptor/toll like receptor family member with regulatory functions in inflammation and immunity [34]. Jia et al. reported that SIGIRR is involved in coronary artery inflammation and coronary endothelial apoptosis in Kawasaki disease [5]. These studies suggest that SIGIRR is capable of inhibiting both inflammation and apoptosis, which is consistent with our results.

With our present data, we conclude that SIGIRR overexpression inhibits caspase-8 activity to alleviate endothelial cell apoptosis and subsequent coronary artery injury in KD. However, some limitations exist in this study. Whether other factors can also mediate caspase-8 activity or SIGIRR can regulate other apoptotic pathways to alleviate coronary artery injury remains uncertain and needs further investigation. Nevertheless, our present data substantiates the good therapeutic efficiency of SIGIRR in KD.

## Conclusion

Our current data demonstrate, for the first time, that SIGIRR has a protective role against KD. SIGIRR up-regulation inhibits endothelial cell apoptosis via suppressing caspase-8, finally mitigating KD coronary artery injury. Since SIGIRR is a receptor protein, designing medications to target this protein would provide more choices for the clinical treatment of KD. Considering that the mainstream treatment for KD is IVIG plus aspirin, which is expensive and not ideal in the face of refractory cases, exploiting small molecules that can up-regulate or activate SIGIRR will also expand the treatment options.

## Data Availability

The datasets obtained in the present study are available from the corresponding author on reasonable request.
